# JMJD3 promotes esophageal squamous cell carcinoma pathogenesis through epigenetic regulation of MYC

**DOI:** 10.1038/s41392-020-00282-9

**Published:** 2020-08-25

**Authors:** Shu-Man Li, Li-Ru He, Jie-Wei Chen, Jie Zhou, Run-Cong Nie, Xiao-Han Jin, Xin Wang, Jian-Hua Fu, Feng-Wei Wang, Dan Xie

**Affiliations:** 1 State Key Laboratory of Oncology in South China, Collaborative Innovation Center for Cancer Medicine, Sun Yat-sen University Cancer Center, Guangzhou, PR China; 2 Department of Medical Oncology, Henan Cancer Hospital, the Affiliated Cancer Hospital of Zhengzhou University, Henan, PR China; 3 Department of Pathology, Sun Yat-sen University Cancer Center, Guangzhou, PR China; 4 Guangdong Esophageal Cancer Institute, Guangzhou, PR China

**Keywords:** Oncogenes, Epigenetics

**Dear Editor**,

Esophageal squamous cell carcinoma (ESCC) is a common cancer worldwide with a 5-year survival rate less than 25%. Reliable molecular markers that could serve as predictors for diagnosis and treatment for ESCC patients are greatly needed. The dysregulation of epigenetic control is one of the common features in cancer. JMJD3 is a histone demethylase specific for H3K27me3/me2 that switches a gene from its repressive state to an active form.^1^ The dysregulation of JMJD3 is reported to play key roles in many cancer progressions. Recently, JMJD3 was reported to be overexpressed in ESCC,^2^ but the potential mechanism of JMJD3 in ESCC remains poorly understood.

Consistent with the literature, both the analysis of the expression of JMJD3 in the TCGA database and our examination in ESCC tissue showed that JMJD3 is significantly overexpressed in ESCC compared to normal tissue (Fig. 1a, Supplementary Fig. 1a–c). The overexpression of JMJD3 was significantly correlated with age, pT status, lymph node metastasis and clinical stage (Supplementary Table 1). Moreover, JMJD3 overexpression was identified as a prognostic factor for ESCC (Supplementary Fig. 1d, e, and Supplementary Table 2). In addition, JMJD3 expression was in negative relationship with the DNA methylation level, and the methylation of CpG island at the promoter of JMJD3 is correlated with better prognosis of ESCC patients (Supplementary Fig. 1f, g).

**Fig. 1:**
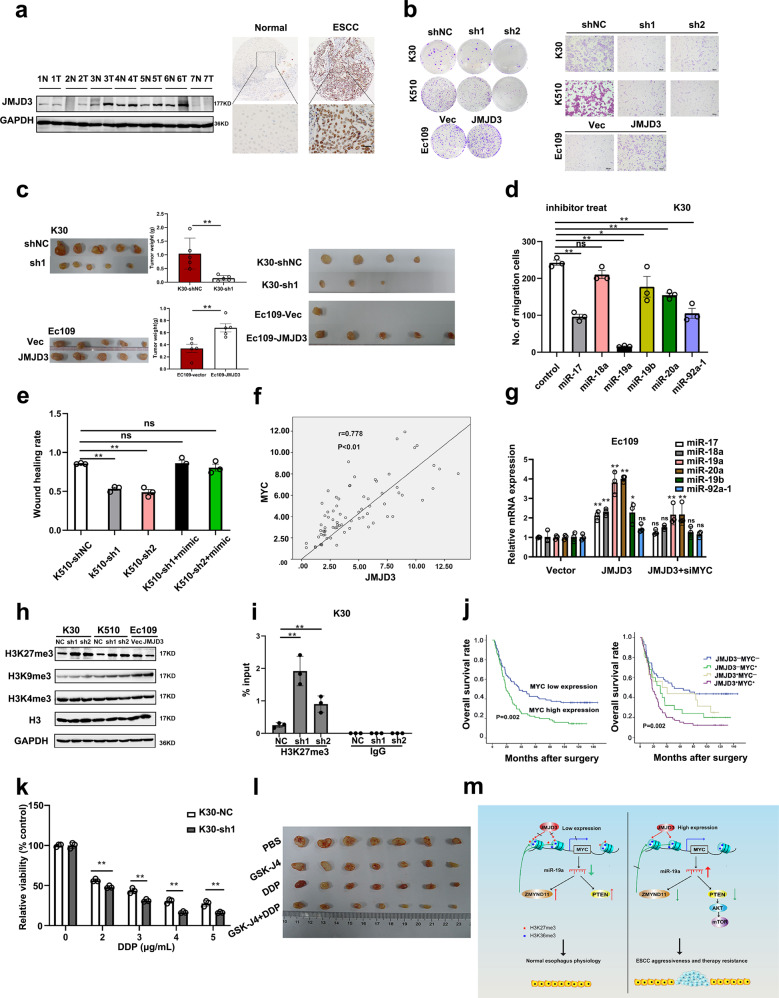
**a** Left panel: the protein levels of JMJD3 in seven pairs of ESCC and adjacent normal esophageal tissues. N normal tissue; T tumor tissue. Right panel: representative IHC images showing the negative expression of JMJD3 in a nonneoplastic esophageal tissue and high expression of JMJD3 in ESCC tissue. Scale bar: 50 μm. **b** Colony formation assay (left panel) and transwell assay (right panel) demonstrated that JMJD3 could significantly promote cell growth and migration ability in vitro. **c** Xenograft tumor model showed that JMJD3 could significantly promote cell growth ability in vivo (left panel), lymph node metastasis assay demonstrated that the number and size of popliteal lymph nodes decreased in JMJD3-silencing cells and increased in JMJD3 ectopic expression group (right panel). **d** Comparison of the effect of miR-17-92 cluster on migration using inhibitor of miRNA. **e** The inhibition of miR-19 could compensate the oncogenic role of JMJD3 in ESCC. **f** The significant positive correlation between MYC and JMJD3 was evaluated in ESCC patients from our hospital. **g** Silencing of MYC by siRNA in Ec109-JMJD3 cells significantly reduced the upregulation of the miR-17-92 cluster. **h** The abundance of H3 lysine methylation was assessed in ESCC cells by WB. **i** ChIP-PCR was performed to assess H3K27me3 occupancy in the MYC promoter. **j** Prognostic significance of MYC and JMJD3 in 161 ESCC patients assessed by Kaplan–Meier analyses. **k** Cell growth assay showed that JMJD3 could confer stronger chemoresistance in ESCC cell lines. **l** Comparison of the antitumor effects of JMJD3 inhibitor (GSK-J4) and chemotherapy (DDP) in vivo. **m** Graphical abstract, the upregulation of JMJD3 and its major mechanism in supporting ESCC cell aggressiveness. The results are expressed as the means ± SD of three independent experiments. [*P < 0.05, **P < 0.01, ns not significant, *t*-test (**c**, **k**), one-way ANOVA (**d**, **e**, **g**, **i**), Pearson correlation test (**f**), log-rank test (**j**)]

To investigate the function of JMJD3 in ESCC cancer cells, stable knockdown of JMJD3 in K30 and K510 cells, and stable JMJD3-transfected Ec109 cells were established (Supplementary Fig. 1h). The knockdown of JMJD3 could significantly suppress cell viability, migration, apoptosis and G0/G1 transition phase in ESCC cells, vice versa in JMJD3-transfected Ec109 cells (Fig. 1b and Supplementary Fig. 2a–g). In addition, we found that GSK-J4, a JMJD3 inhibitor, could suppress cell growth rate and cell migration (Supplementary Fig. 2h, i). Further, the in vivo experiments showed that JMJD3 promoted ESCC tumor growth (Fig. 1c, left) and lymph node metastasis (Fig. 1c, right). Moreover, JMJD3 silencing in ESCC cells led to a reduced proportion of the stemness markers and SP cells (Supplementary Fig. 2j, k). Tumor formation assay showed an increased tumorigenicity in JMJD3 ectopic expression cells, vice versa in JMJD3 silencing cells (Supplementary Fig. 2l).

Accumulating evidences indicated that microRNAs (miRNAs) were promising tools and targets for therapy in disease. To explore the mechanism of JMJD3 oncogenic effects on ESCC, human miRNA microarray was performed. MiRNA microarray showed that the miR-17-92 cluster, known as “oncomiR-1”, was down-regulated after knockdown the expression of JMJD3, the result of which was further confirmed by RT-qPCR (Supplementary Fig. 3a, b). Moreover, TCGA database analysis showed that expression of miR-17-92 cluster gene (MIR17HG) in esophageal cancer is much higher, and high expression of MIR17HG is correlated with poor survival of ESCC patient (Supplementary Fig. 3c).

We further investigated the role of each member of the miR-17-92 cluster in mediating JMJD3 function with their inhibitors via performing transwell, CCK-8 and apoptosis assay. The results indicated that miR-19a exhibited the most remarkable suppressive effect on malignant phenotypes of ESCC (Supplementary Fig. 3d–f). Moreover, miR-19a was upregulated in ESCC tissues (Supplementary Fig. 3g), and its expression was positively correlated with the mRNA expression level of JMJD3 (Supplementary Fig. 3h). Rescue experiments showed that miR-19a mediated at least partly the oncogenic effect of JMJD3 (Fig. 1d, e and Supplementary Fig. 4a–d). Two targets of miR-17-92, PTEN and ZMYND11 (Supplementary Table 3), were also found to be regulated by JMJD3 (Supplementary Fig. 5a). Furthermore, ectopic JMJD3 expression could activate PTEN/AKT/mTOR pathway, which could be blocked by inhibition of miR-19a (Supplementary Fig. 5b).

Previous studies have shown that miR-17-92 is regulated by MYC.^3^ Our findings showed that JMJD3 could significantly promote the mRNA and protein expression of MYC (Supplementary Fig. 5c, d), and MYC expression was positively correlated with JMJD3 expression at the mRNA levels in ESCC tissues (Fig. 1f and Supplementary Fig. 5e). Downregulation of MYC could ablate the upregulation of miR-17-92 cluster by JMJD3 (Fig. 1g), which further supports the involvement of MYC in mediating the function of JMJD3. Interestingly, ZMYND11 has been reported to interact with MYC as a tumor suppressor,^4^ which further supports the involvement of ZMYND11 in JMJD3/ MYC/miR-17-92 axis.

Among histone H3K4me3, H3K9me3, and H3K27me3, only H3K27me3 modification was affected by changes in JMJD3 expression (Fig. 1h). To explore the mechanism how JMJD3 regulates MYC expression, promoter luciferase reporter assay was performed. JMJD3 knockdown could significantly suppress the fluorescence intensity of MYC promoter and mainly through the −873 to −1043 bp region of the promoter, which indicates that JMJD3 promotes MYC expression through its promoter (Supplementary Fig. 5f). JMJD3 is a histone demethylase reversing methylation of histone at gene promoter, which could often lead to changes in gene activity.^5^ We speculated that JMJD3 regulates MYC promoter transcriptional activity through H3K27me3. To further investigate this hypothesis, ChIP-PCR was carried out using H3K27me3 antibody to analyze the binding intensity of H3K27me3 on the promoter of MYC (Fig. 1i). Our result indicated that JMJD3 could affect H3K27me3 of MYC promoter via regulating the methylation of H3k27, and enhance MYC transcriptional activity.

Moreover, the expressions of MYC and JMJD3 were also examined simultaneously by IHC in the ESCC TMA (Supplementary Fig. 5h). The frequency of cases with high expression of MYC was significantly higher in ESCC that exhibited high expression of JMJD3 than in those cases with low expression of JMJD3 (Supplementary Table 4). As expected, high expression of MYC was significantly correlated with poor survival of ESCC patients (Fig. 1j, left). ESCC patients with high expression of both MYC and JMJD3 exhibited the shortest survival rate, whereas patients with low expression of both MYC and JMJD3 demonstrated the most favorable survival (Fig. 1j, right panel).

Since JMJD3 could enhance the stemness-like traits of ESCC cells, we speculate that JMJD3 might be closely correlated with therapy resistance of ESCC. Cell growth assay revealed that JMJD3 could increase cell viability of ESCC cells after treatment with DDP or 5-FU (Fig. 1k and Supplementary Fig. 6a). In the colony formation assay, after X-ray radiation, JMJD3-expressed cells formed more colonies than the matched control cells, whereas JMJD3 knockdown cells had fewer colonies compared to the vector-carrying cells (Supplementary Fig. 6b). The in vivo mouse model indicated that GSK-J4 could significantly suppress the tumor growth of ESCC cells and enhance the antitumor effect of DDP (Fig. 1l and Supplementary Fig. 6c).

We further performed IHC in the ESCC patients treated with CRT therapy to validate the association of JMJD3 expression and CRT response. Notably, high expression of JMJD3 was observed more frequently in non-CR group than in CR group (Supplementary Table 5). These results indicated that JMJD3 confers therapy resistance in ESCC cells.

Herein, we proposed a molecular model for JMJD3 in ESCC cell aggressiveness and therapy resistance (Fig. 1m). Our data suggest that JMJD3 plays an important role in the progression of ESCC through the JMJD3/MYC/miR-17-92 pathway to affect cell proliferation, metastasis, apoptosis, stemness-like traits, and sensitivity to therapy. JMJD3 was reported to be a tumor suppressor by interacting with proteins such as p53 and Rb. However, p53 and its accessory protein ATG7 were highly mutated (80%) and deleted in ESCC, respectively. These evidences might partly explain why JMJD3 could bypass the reported tumor suppressor mechanism in ESCC. In conclusion, JMJD3 might serve as a prognostic biomarker and a therapy target for ESCC.

## Supplementary information

supplemental materials

## Data Availability

All the data and materials in this paper could be found in the RDD database of our hospital. Our RDD number is RDDB2020000953 (www.researchdata.org.cn). The miRNA microarray data were uploaded to Gene Expression Omnibus (GEO) database (GSE142007).
