# Supplementary material for: JMJD3 promotes esophageal squamous cell carcinoma pathogenesis through epigenetic regulation of MYC
Source: Signal Transduct Target Ther. 2020 Aug 25;5:165. doi: 10.1038/s41392-020-00282-9 (PMC7447641; doi:10.1038/s41392-020-00282-9)
Supplement: Supplementary file 1 — supplemental materials [file 41392_2020_282_MOESM1_ESM.docx]

**Supplemental Materials**

**JMJD3 promotes esophageal squamous cell carcinoma** **pathogenesis through epigenetic regulation of MYC**

Shu-Man Li^1, 2#^, Li-Ru He^1#^, Jie-Wei Chen^1, 3#^, Jie Zhou^1#^, Run-Cong Nie^1#^, Xiao-Han Jin^1^, Xin Wang^1^, Jian-Hua Fu^1, 4^, Feng-Wei Wang^1*^, Dan Xie^1, 3*^

^1^ State Key Laboratory of Oncology in South China; Collaborative Innovation Center for Cancer Medicine; Sun Yat-sen University Cancer Center, Guangzhou, P. R. China.

^2^ Department of Medical Oncology, Henan Cancer Hospital, the Affiliated Cancer Hospital of Zhengzhou University, Henan, P. R. China.

^3^ Department of Pathology, Sun Yat-sen University Cancer Center, Guangzhou, P. R. China.

^4^ Guangdong Esophageal Cancer Institute, Guangzhou, P. R. China.

^#^ These authors contributed equally to this article.

***Corresponding Author:** Dr. Dan Xie or Dr. Feng-Wei Wang, State Key Laboratory of Oncology in South China, Cancer Center, Sun Yat-sen University, No. 651, Dongfeng Road East, Guangzhou 510060, Guangdong, P. R. China. Telephone number: 86-20-87343193; Fax: 86-20-87343170; E-mail: xiedan@sysucc.org.cn (D.X.) or wangfengw@sysucc.org.cn (F.W.W.).

**This file includes:**

Supplemental Figures 3

Fig. S1. JMJD3 is upregulated in ESCC 3

Fig. S2. JMJD3 promotes ESCC pathogenesis 5

Fig. S3. miR-17-92 cluster is upregulated in ESCC 8

Fig. S4. The oncogenic function of JMJD3 is mediated by miR-17-92 cluster 10

Fig. S5. JMJD3 regulated miR-17-92 cluster through MYC by epigenetic modifications 11

Fig. S6. JMJD3 confers therapy resistance in ESCC cells 13

Tables 15

Materials and Methods 22

References 28

**
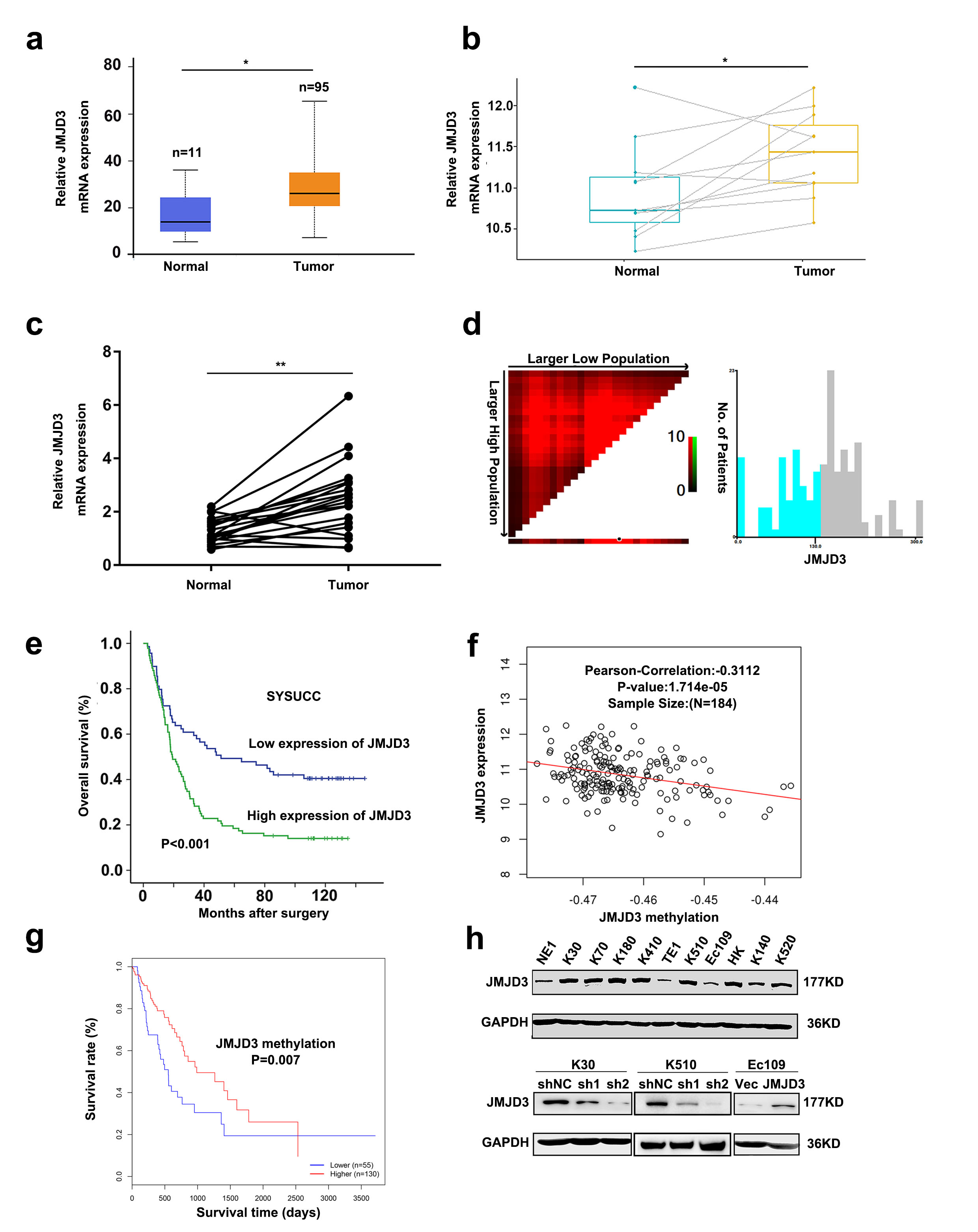
**

**Figure S1.** **JMJD3 is upregulated in ESCC**. (a) The relative expression of JMJD3 in normal esophageal tissues and ESCC tissues from the TCGA database. (b) JMJD3 mRNA in paired ESCC tissues from the TCGA database (n = 11), of these tissues, 9/11(81.8%) is upregulated in ESCC compared with their paired normal tissues. (c) Relative expression of JMJD3 detected by RT-qPCR in 20 ESCC tissues compared with their matched non-tumor tissues. (d) Cutoff value of JMJD3 in this analysis was determined by ROC curve analysis. (e) Kaplan–Meier analysis indicating the relationship between high expression of JMJD3 and poor overall survival in ESCC. (f) Cutoff value of JMJD3 in this analysis was determined as 130 by ROC curve analysis. (f) The mRNA expression of JMJD3 was in negative relationship with the DNA methylation level. (g) The methylation of CpG island at the promoter of JMJD3 is correlated with better prognosis of ESCC patients. (h) Upper panel, the protein levels of JMJD3 were analyzed by WB in ten human ESCC cell lines and an immortalized normal esophageal epithelial cell line NE1. Lower panel, the protein levels of JMJD3 was detected by WB in ESCC cell lines treated with JMJD3 shRNAs or ectopic expression of JMJD3 in JMJD3-transfected cells. (**P* < 0.05, ***P* < 0.01, *t*-test [a], paired *t*-test [b, c], log-rank test [e, g]).

**
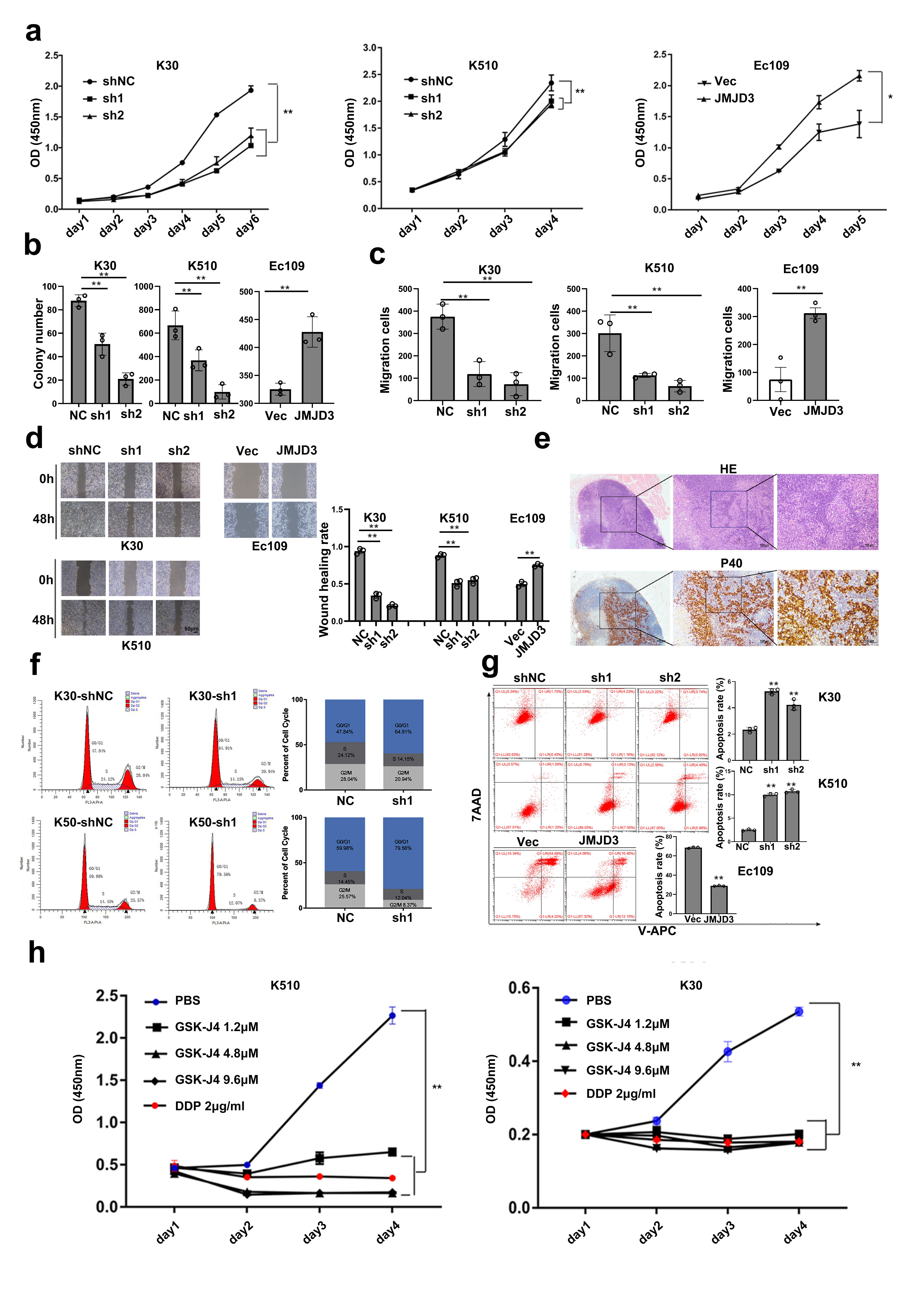
**

**
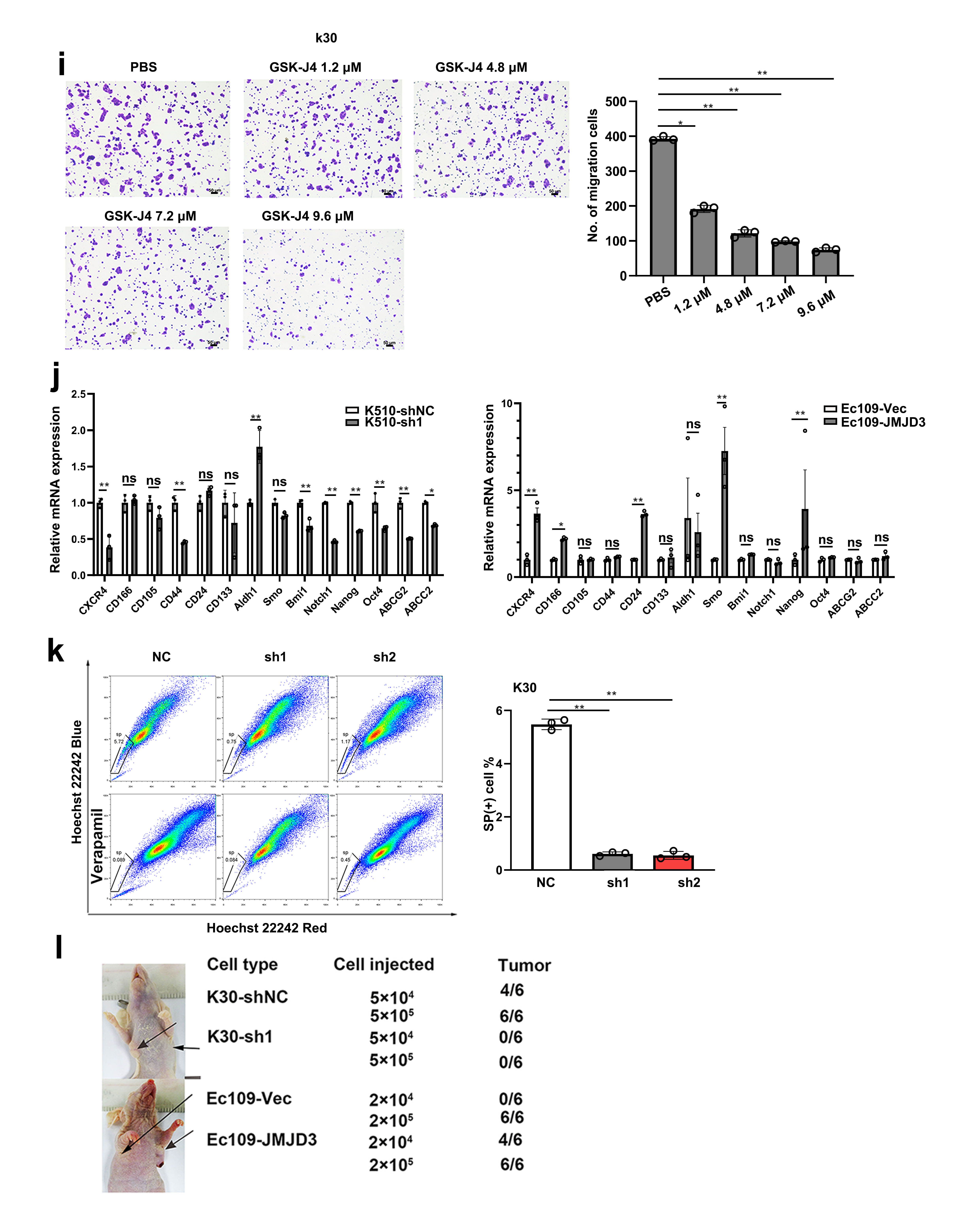
**

**Figure S2. JMJD3 promotes ESCC pathogenesis.** Cell growth assay **(**a), Colony formation assay (b), transwell assay (c) and wound-healing assay (d) demonstrated that JMJD3 could significantly promote cell growth ability and migration ability *in vitro*. (e) The representative pictures of HE and ESCC maker P40 in popliteal lymph nodes. Scale bar: 100 μm. (f) The percentage of G0/G1phase increased after silencing of JMJD3 in ESCC cells, cell cycle distributions were analyzed by flow cytometry. (g) JMJD3 could confer resistance to apoptosis, the cell apoptosis rate was determined by cytometry ESCC cell lines. Staurosporine (Selleck, S1421, 1 μmol/L) was used to induce apoptosis in Ec109 cell lines. (h-i) JMJD3 inhibitor GSK-J4 suppressed cell growth and cell migration with PBS (phosphate buffer saline) as negative control and DDP as positive control. (j) Relative expression of stemness-associated genes (Nanog, Oct-4, Bmi-1, Notch-1, and Smo), multiple drug-resistant transporter genes (ABCC2, ABCG2) and surface antigens associated with cancer stem cells (CD24, CD44, CD133, CD105, and CD166) were compared by RT-qPCR between JMJD3 overexpression and silencing ESCC cells. (k) JMJD3 substantially upregulated the proportion of SP cells. (l) Tumor formation in nude mice showed increased *in vivo* tumorigenicity in Ec109-JMJD3 group and decreased tumorigenicity in JMJD3 silencing group compared with control group. (The results are expressed as the means ± SD, **P* < 0.5, ***P* < 0.01, ns [not significant], *t*-test [j], one-way ANOVA [a-d, g-i, k]).

**
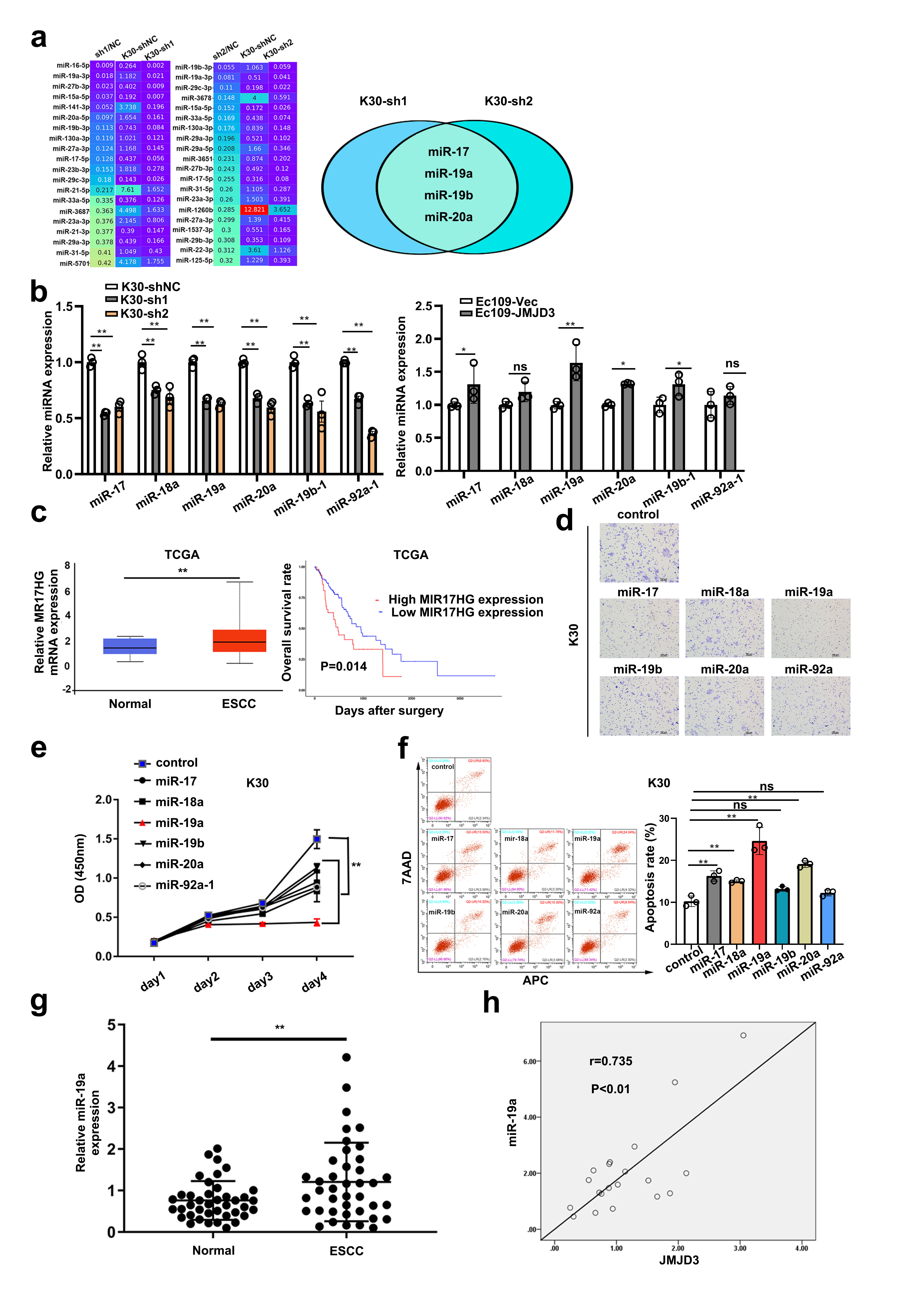
**

**Figure S3. miR-17-92 cluster is upregulated in ESCC**. Top 20 down regulated miRNAs in JMJD3-silence cell lines by miRNA microarray and the members of miR-17-92 cluster changed in both cell lines. (b) Expression level of miR-17-92 cluster was verified in ESCC cell line K30 and Ec109 by RT-qPCR. (c) The expression of MIR17HG in esophageal cancer tissues and normal esophageal in the TCGA database, and the relationship between high expression of MIR17HG and poor overall survival in ESCC. (d-f) Comparison of the effect of miR-17-92 cluster on migration, proliferation, and apoptosis resistance using inhibitor of miRNA. (g) Relative expression of miR-19a in 40 ESCC tissues and their matched normal tissues by RT-qPCR. (h) Pearson correlation analysis showed there was a positive correlation between miR-19a and JMJD3 in the mRNA level in 20 ESCC samples from our hospital. (The results are expressed as the means ± SD, **P* < 0.5, ***P* < 0.01, ns [not significant], *t*-test [c left, g], one-way ANOVA [b, e, f], log-rank test [c right], Pearson correlation test [h]).

**
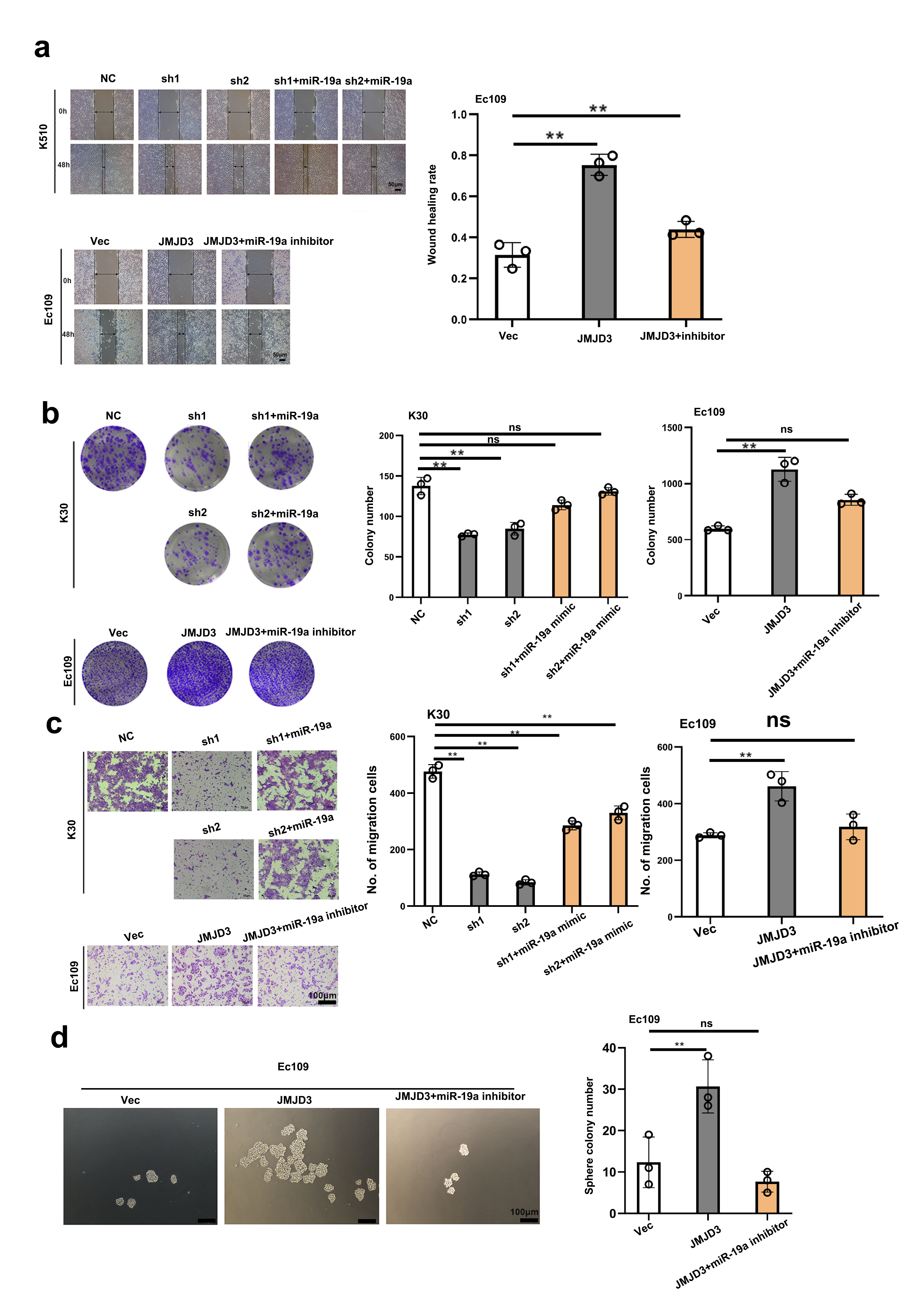
**

**Figure S4.** **The oncogenic function of JMJD3 is mediated by miR-17-92 cluster**. (a-d) The inhibition of miR-19 could compensate the oncogenic role of JMJD3 in ESCC. The results are expressed as the means ± SD of three independent experiments. (**P* < 0.05, ***P* < 0.01, ns [not significant], one-way ANOVA [a-d]).

**
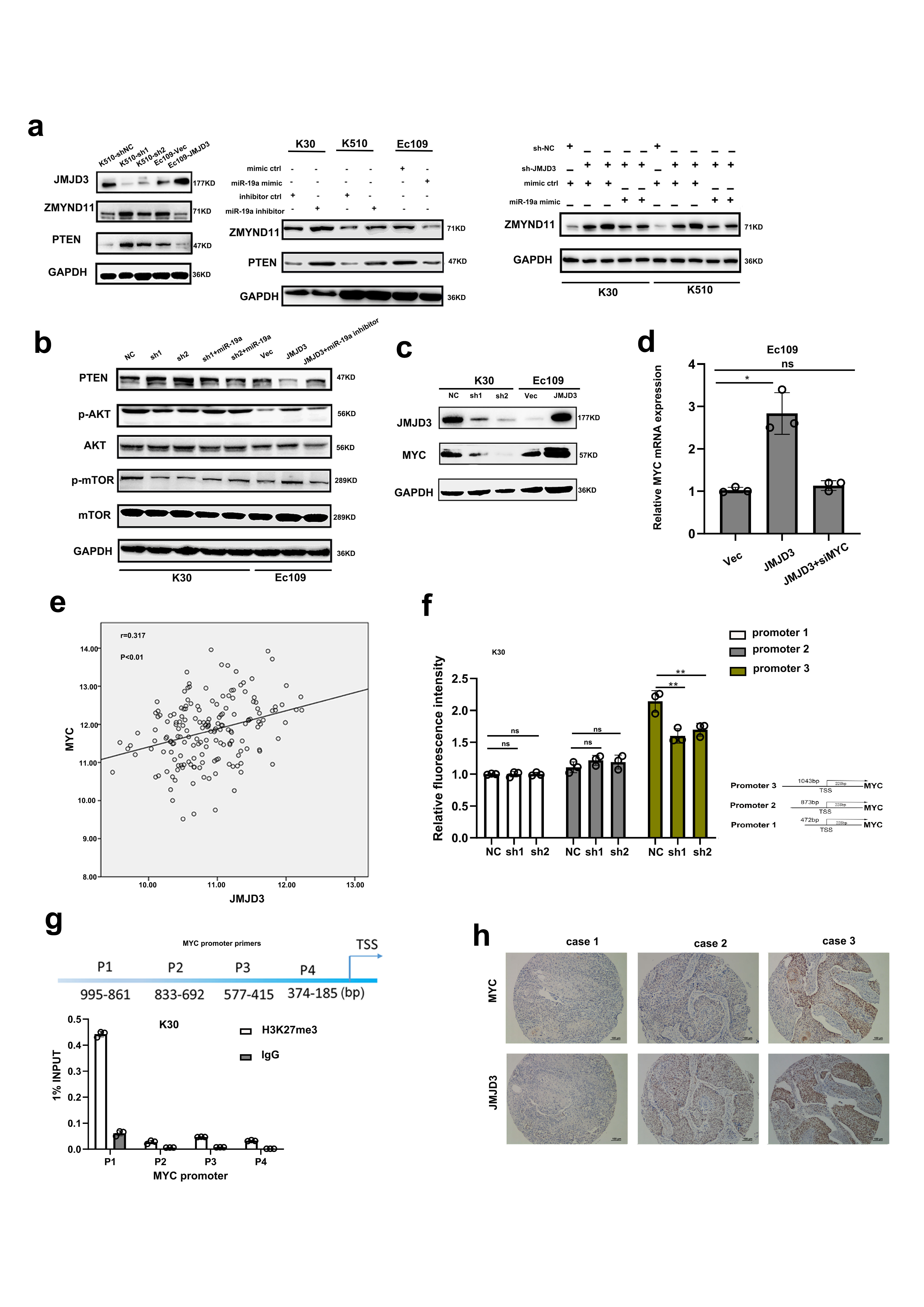
**

**Figure S5. JMJD3 regulated miR-17-92 cluster through MYC by epigenetic modifications.** (a) The expression of JMJD3, ZMYND11 and PTEN in the indicated ESCC cell lines. (b) The expressions of PTEN/AKT/mTOR in the indicated cells were measured by WB. (c-d) WB and PCR showed that the expression level of MYC changed along with the JMJD3 expression. (e) The significant positive correlation between MYC and JMJD3 was evaluated in ESCC patients from TCGA. (f) Promoter reporter assay was performed to confirm the domain of MYC promoter mediating the effect of JMJD3 on MYC expression. (g) The schematic diagram of four pairs primer of MYC primers for the ChIP assay. P1 was selected for further experiments. (h) Expressions of MYC and JMJD3 were examined by IHC in the same ESCC TMA (161 cases). Scale bar: 100 μm. (**P* < 0.5, ***P* < 0.01, ns [not significant], one-way ANOVA [d, f], Pearson correlation test [e]

**
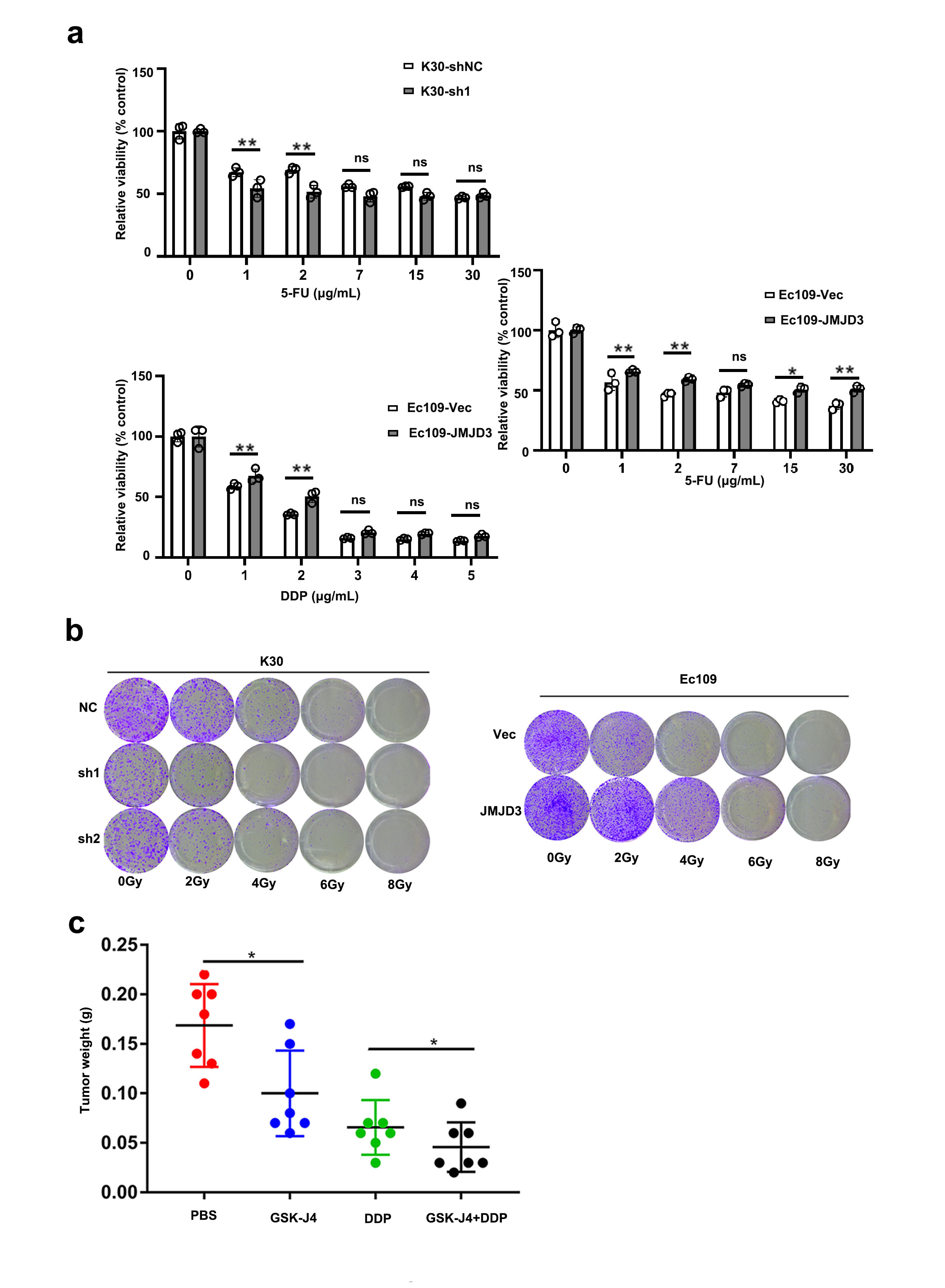
Figure S6. JMJD3 confers therapy resistance in ESCC cells.** (a) Cell growth assay showed that JMJD3 could confer stronger chemoresistance in ESCC cell lines. The results are expressed as the mean ± SD of three independent experiments (**P* <0.05, ***P*<0.01, ns [not significant], *t*-test). (b) Colony forming assay showed that JMJD3 could confer stronger radioresistance in ESCC cell lines. (c) Compare the antitumor effects of JMJD3 inhibitor (GSK-J4), chemotherapy (Cisplatin) in vivo. The values are expressed as the mean ± SD of seven mice. **P*<0.05, ANOVA with post hoc test.

**Supplementary Table 1. Correlation of JMJD3 expression with patients’ clinicopathological features in primary esophageal squamous cell carcinomas**

| **Variables** | **JMJD3 expression** | | | |
| --- | --- | --- | --- | --- |
|  | **Cases** | **Absent** | **Present** | ***P* value*** |
| Age (years) |  |  |  | 0.005 |
| ≤ 59.0^†^ | 87 | 46 (52.9%) | 41 (47.1%) |  |
| > 59.0 | 74 | 23 (31.1%) | 51 (68.9%) |  |
| Gender |  |  |  | 1.000 |
| Female | 28 | 12 (42.9%) | 16 (57.1%) |  |
| Male | 133 | 57 (42.9%) | 76 (57.1%) |  |
| Location |  |  |  | 0.775 |
| Upper | 18 | 9 (50.0%) | 9 (50.0%) |  |
| Middle | 102 | 42 (41.2%) | 60 (58.8%) |  |
| Lower | 41 | 18 (43.9%) | 23 (56.1%) |  |
| Tumor size (cm) |  |  |  | 0.250 |
| ≤ 5^‡^ | 92 | 43 (61.5%) | 49 (38.5%) |  |
| > 5 | 69 | 26 (53.1%) | 43 (46.9%) |  |
| Differentiation |  |  |  | 0.068 |
| Well | 29 | 17 (58.6%) | 12 (41.4%) |  |
| Moderate | 82 | 36 (43.9%) | 46 (56.1%) |  |
| Poor | 50 | 16 (32.0%) | 34 (68.0%) |  |
| pT status |  |  |  | 0.002 |
| T1-2 | 27 | 19 (76.5%) | 8 (23.5%) |  |
| T3-4 | 134 | 50 (66.7%) | 84 (33.3%) |  |
| pN status |  |  |  | 0.033 |
| N0 | 41 | 21 (51.2%) | 20 (48.8%) |  |
| N1 | 48 | 23 (47.9%) | 25 (52.1%) |  |
| N2 | 50 | 20 (40.0%) | 30 (60.0%) |  |
| N3 | 22 | 5 (22.7%) | 17 (77.3%) |  |
| Stage |  |  |  | 0.005 |
| I | 117 | 58 (49.6%) | 59 (50.4%) |  |
| II-III | 44 | 11 (25.0%) | 33 (75.0%) |  |

*Chi-square test; ^†^Median age; ^‡^Median size.

**Supplementary Table 2. Cox multivariate analyses of prognostic factors on overall survival**

| **Variables** | **Hazard ratio** | **95% CI** | ***P* value** |
| --- | --- | --- | --- |
| pT status (T1-2 *v* T3-4) | 1.986 | 1.050-3.757 | 0.035 |
| pN status (N0-1 *v* N2-3) | 1.553 | 1.071-2.253 | 0.020 |
| JMJD3 expression (low *v* high) | 1.682 | 1.134-2.496 | 0.010 |
| CI, confidence interval. |  |  |  |

**Supplementary Table 3. The predicted target genes of miR-19a**

| TaregetScan7.1filtered | | |  | mirdbV5filtered | |  |
| --- | --- | --- | --- | --- | --- | --- |
| **miRNAname** | **targetGene** | **cumulativeWeightedContext++Score** | **Score** | **miRNAname** | **targetGene** | **targetScore** |
| hsa-miR-19a-3p | KBTBD8 | -1.2 | -1.2 | hsa-miR-19a-3p | ATP6V1B2 | 100 |
| hsa-miR-19a-3p | ZMYND11 | -1.06 | -1.1 | hsa-miR-19a-3p | ZMYND11 | 100 |
| hsa-miR-19a-3p | CHIC1 | -0.98 | -1.01 | hsa-miR-19a-3p | SLC35F1 | 100 |
| hsa-miR-19a-3p | C11orf96 | -0.96 | -1.16 | hsa-miR-19a-3p | LRP2 | 100 |
| hsa-miR-19a-3p | QKI | -0.88 | -0.95 | hsa-miR-19a-3p | CLOCK | 100 |
| hsa-miR-19a-3p | PMEPA1 | -0.87 | -0.87 | hsa-miR-19a-3p | SECISBP2L | 100 |
| hsa-miR-19a-3p | LONRF1 | -0.84 | -0.84 | hsa-miR-19a-3p | LONRF1 | 99 |
| hsa-miR-19a-3p | ZBTB18 | -0.83 | -0.84 | hsa-miR-19a-3p | MPPED2 | 99 |
| hsa-miR-19a-3p | RNF11 | -0.81 | -1.11 | hsa-miR-19a-3p | SPTSSB | 99 |
| hsa-miR-19a-3p | HBP1 | -0.78 | -0.78 | hsa-miR-19a-3p | WDR44 | 99 |
| hsa-miR-19a-3p | KCNA4 | -0.76 | -0.76 | hsa-miR-19a-3p | IGFBP3 | 99 |
| hsa-miR-19a-3p | NEUROD1 | -0.76 | -0.86 | hsa-miR-19a-3p | CACUL1 | 99 |
| hsa-miR-19a-3p | SPTSSB | -0.74 | -0.74 | hsa-miR-19a-3p | CHIC1 | 99 |
| hsa-miR-19a-3p | DTNA | -0.74 | -0.75 | hsa-miR-19a-3p | CDS1 | 99 |
| hsa-miR-19a-3p | MPPED2 | -0.73 | -0.73 | hsa-miR-19a-3p | SOX6 | 99 |
| hsa-miR-19a-3p | DNAJA2 | -0.72 | -0.74 | hsa-miR-19a-3p | RPS6KA5 | 99 |
| hsa-miR-19a-3p | TAOK1 | -0.7 | -0.79 | hsa-miR-19a-3p | CCNL1 | 99 |
| hsa-miR-19a-3p | PLA2G10 | -0.7 | -0.7 | hsa-miR-19a-3p | SYT1 | 99 |
| hsa-miR-19a-3p | CDS1 | -0.69 | -0.75 | hsa-miR-19a-3p | E2F8 | 99 |

| **Supplementary Table 4. Association of JMJD3 expression and MYC expression in ESCC** | | |
| --- | --- | --- |
| JMJD3 expression Cases | MYC expression | ***P*** value |
|  | Low (%) High (%) |  |
| Low 69 | 55 (79.7) 14 (20.3) | < 0.001 |
| High 92 | 25 (27.2) 67 (72.8) |  |
| Chi-square test; |  |  |

|  |
| --- |

| **Supplementary Table 5. Association of JMJD3 expression and CRT response** | | |
| --- | --- | --- |
| JMJD3 expression Cases | CRT response | ***P*** value |
|  | CR No CR |  |
| Low 97 | 33 (34.0) 64 (66.0) | < 0.001 |
| High 61 | 6 (9.8) 55 (90.2) |  |
| Chi-square test; |  |  |
|  |  |  |

| **Supplementary Table 6. Antibodies used in this study** | | |
| --- | --- | --- |
| **Antibody** | **Brand** | **Catalog number** |
| JMJD3 | abcam | WB ab85392; IHC ab38113 |
| MYC | abconal | A0661 |
| PTEN | proteinteck | 22034-1-AP |
| ZMYND11 | abcam | ab190890 |
| AKT | CST | 4691 |
| P-AKT | CST | 4060 |
| mTOR | CST | 2983T |
| p-mTOR | CST | 5536T |
| GAPDH | proteinteck | 1E6D9 |
| H3 | abclonal | A2348 |
| H3K4me3 | abconal | A2357 |
| H3K9me3 | abconal | A2360 |
| H3K27me3 | abconal | WB A2363, ChIP CST9733s |

| **Supplementary Table 7. Primers used in this study** | | |
| --- | --- | --- |
| **Gene name** | **Forward (5'to3')** | **Reverse (5'to3')** |
| Oct-4 | CTTGCTGCAGAAGTGGGTGGAGGAA | CTGCAGTGTGGGTTTCGGGCA |
| Nanog | AATACCTCAGCCTCCAGCAGATG | TGCGTCACACCATTGCTATTCTTC |
| Notch1 | CCTGAGGGCTTCAAAGTGTC | CGGAACTTCTTGGTCTCCAG |
| Bmi1 | TGGAGAAGGAATGGTCCACTTC | GTGAGGAAACTGTGGATGAGGA |
| Smo | TGGTCACTCCCCTTTGTCCTCAC | GCACGGTATCGGTAGTTCTTGTAGC |
| Aldh1 | TTGGAATTTCCCGTTGGTTA | CTGTAGGCCCATAACCAGGA |
| CD133 | TGGATGCAGAACTTGACAACGT | ATACCTGCTACGACAGTCGTGGT |
| CD24 | TGAAGAACATGTGAGAGGTTTGAC | GAAAACTGAATCTCCATTCCACAA |
| CD44 | TTGCAGTCAACAGTCGAAGAAG | CCTTGTTCACCAAATGCACCA |
| CD105 | CGCCAACCACAACATGCAG | GCTCCACGAAGGATGCCAC |
| CD166 | TCCTGCCGTCTGCTCTTCT | TTCTGAGGTACGTCAAGTCGG |
| CXCR4 | ACTACACCGAGGAAATGGGCT | TTCTTCACGGAAACAGGGTTC |
| PTEN | TAAGGACCAGAGACAAAAAGGGA | GGCAGACCACAAACTGAGGATT |
| MYC | CGTCCTCGGATTCTCTGCTC | CGATTTCTTCCTCATCTTCTTGTTC |
| JMJD3 | ACGAGTCAGAGCACGATAGTGAG | GAGCCAGTATGAAAGTTCCAGAG |
| GAPDH | GGACCTGACCTGCCGTCTAG | GTAGCCCAGGATGCCCTTGA |
| ABCC2 | ATGCAGCCTCCATAACCATGA | CTTCGTCTTCCTTCAGGCTATTCA |
| ABCG2 | TCATCAGCCTCGATATTCCATCT | GGCCCGTGGAACATAAGTCTT |
| MYC-P1 | GAGACTGTTGCAAACCGGCGCCACA | TTGCCTGCTCTCTGCCAGTCTGTA |
| MYC-P2 | AACAGGCAGACACATCTCAGGGCTAAA | ATTGGATACCTTCCACCCAGA |
| MYC-P3 | TAATCATTCTAGGCATCGTT | GCCTCGCTAAGGCTGGGGAAA |
| MYC-P4 | CAGTGCGTTCTCGGTGTGGA | ATGTAAACAGAGTAAGAGAGCCGC |

**Materials and Methods**

**ESCC cell lines and tissue samples**

In this study, the data of 161 ESCC patients who were treated at the Sun Yat-sen University Cancer Center (SYSUCC) between April 2005 and December 2010 were investigated. Cases were selected based on ESCC pathological diagnosis, history of primary and curative resection for ESCC, availability of resection tissue, and follow-up data. Tissue microarray (TMA) was constructed as previously described ^1^. Another 158 cases of ESCC treated with definitive CRT between April 2000 and June 2012 were retrieved from the Department of Radiotherapy (SYSUCC). These cases were selected based on previously described criteria ^2^. ESCC patients who previously received chemotherapy and/or radiotherapy were excluded. The paired fresh ESCC tumor tissues used for western blot (WB) and quantitative reverse transcription PCR (RT-qPCR) were obtained from the tumor resource bank of SYSUCC. ESCC cell lines, including K30, K70, K180, K140, K410, K510, K520, TE1, Ec109, and HK were cultured in Dulbecco’s modified Eagle medium (Gibico, CA) supplemented with 10% fetal bovine serum (HyClone, Logan, UT), and normal immortalized esophageal cell line NE1 was propagated in defined keratinocyte serum-free medium (K-SFM; Gibco, CA)

**Immunohistochemistry (IHC)**

IHC and cutoff score determination were performed as previously described ^3^.

**Plasmid constructs and transfection**

Full-length human JMJD3 complementary DNA (cDNA) was cloned into pReceiver-M12 vector (GeneCopoeia, Guangzhou, China). Lentiviral psi-LVRU6GP containing short hairpin RNAs (shRNA) that target JMJD3 was purchased from GeneCopoeia (Guangzhou, China). Cells transfected with empty vector were used as controls. miR-17-92 mimic, miR-17-92 inhibitor, and the siRNAs specifically against MYC and ZMYND11 were all purchased from Ribobio (Guangzhou, China). The transfection was performed by Lipofectamine 2000 according to the manufacturer’s instructions.

**MicroRNA (miRNA) microarray and data analysis**

Total RNA was extracted from ESCC cell lines with TRIzol reagent (Invitrogen, CA). The miRNA microarray analysis was carried out by KangChen Biotech (Shanghai, China) by following the manufacturer's instructions. RNA labeling and array hybridization were performed by following Exiqon's manual. GenePix Pro 6.0 software (Axon) was applied to generate raw data. The expressed data were normalized using the Median normalization. The threshold value for significant upregulation or downregulation of miRNAs was a fold change ≥1.5 with a value of *P* < 0.05, as calculated by *t*-test.

**WB analysis**

WB was carried out in compliance with the standard protocol.

**RNA isolation and** **RT-qPCR**

Total RNA was extracted from ESCC tissues and cell lines using TRIzol. RT-qPCR was performed using SYBR Green SuperMix (Roche) and ABI7900HT Fast Real-Time PCR system (Applied Biosystems).

**Cell growth assay**

Cell viability was measured using the CCK-8 assay (Dojindo). After starvation treatment, cells were seeded into 96-well plates (1 × 10^3^ cells/well). Cell viability was measured by following the manufacturer’s instructions.

**Xenograft tumor growth and tumor formation assay**

In xenograft tumor growth assay, four-week-old nude mice were randomly divided into different groups (n = 5). Each mouse was injected with 2 × 10^6^ K30-shNC or K30-shJMJD3, Ec109-Vector or Ec109-JMJD3 cells via subcutaneous injection. In tumor formation assay, different amounts of K30-shNC or K30-shJMJD3, Ec109-Vector or Ec109-JMJD3 cells were injected via subcutaneous injection. Six weeks after the injection, the mice were sacrificed and examined. All mice used for the *in vivo* studies were female BALB/c nude strains (CAnN. Cg-Foxn1nu/Crl), purchased from Charles River (Beijing, China), between 4-12 weeks old (according to the experimental design), and weighed 15-30 ± 3-5 g.

**Lymph node metastasis** **model**

In the lymph node metastasis model, 5 × 10^5^ cells were injected into mouse paw (n = 5). After 8-12 weeks, the mice were sacrificed and their popliteal lymph nodes, which represent the sentinel lymph node for the model, were examined ^4^. Swollen popliteal lymph nodes were observed and lymph node metastasis was determined by haematoxylin and eosin (HE) staining in all the swollen popliteal lymph nodes.

**Drug sensitivity assay**

1000 ESCC cells were seeded into 96-well plates overnight, and then different concentrations of cisplatin (DDP) or 5-fluorouracil (5-FU) were added and incubated for another 48 hours, followed by cell sensitivity determination using the CCK-8 assay.

**X-ray sensitivity assay**

ESCC cells were seeded into 6-well plates (3 × 10^2^ cells/well). Different doses of X-ray were used to treat the cells for 24 hours. Then the cells were cultured for 2 weeks and the clone number was counted.

**Luciferase reporter assay**

The luciferase reporter assay was performed using the Dual-Luciferase Reporter Assay System (Promega, Madison, WI). Three MYC promoters of various lengths were cloned into pEZX-PG04 plasmid. ESCC cells were then transfected with the pEZX-MYC-promoter1, pEZX-MYC-promoter2 or pEZX-MYC-promoter3. All reporter gene assays were performed with a dual luciferase assay kit (Promega) according to the manufacturer’s instructions.

**Chromatin immunoprecipitation (ChIP) assay**

ChIP assay was performed as previously described ^5^ using specific antibody against H3K27me3 (1:50; CST 9733s). The enrichment of specific regions in the promoter was assessed by PCR amplification in a 10 μL reaction system.

**Side population (SP) assay**

ESCC cells (1 × 10^6^) were incubated with Hochest3342 with or without verapamil for 90 min at 37°C, and the SP population rate was detected by the cytometry.

**Statistical analysis**

Clinical correlations were analyzed by Pearson correlation test and survival analyses were assessed by Kaplan-Meier plots and log-rank tests. Univariate and multivariate survival analyses were performed by Cox proportional hazards regression model. The measurement data were expressed by means ± SD. The *t*-test was performed for comparison between two groups, and one-way ANOVA was used for comparison among multiple groups. SPSS 20.0 and GraphPad Prism 7 were used for data analyses. Results were considered statistically significant when *P* < 0.05.

**Ethics statement**

All experiments were approved by the Research Ethics Committee of SYSUCC (No. YB2018-008). All the animal experiments were performed in accordance with relevant guidelines and regulations. No informed consent was obtained since most of the patients in this study were dead; the Ethics Committee waived the need for consent. All samples were anonymous.

**Acknowledgements**

This study was supported by the grants from the National Key R&D Program of China (Nos. 2017YFC1309001), the National Natural Science Foundation of China (Nos. U1601229, 81572359, 81430055, 81602063 and 81730072), the Natural Science Foundation of Guangdong (No. S2014030001589), the Doctor start-up project of Natural Science Foundation of Guangdong (2016A030310231), the Program for Changjiang Scholars and Innovative Research Team in University of Ministry of Education of China (No. IRT-15R13) and Guangdong Esophageal Cancer Institute Science and Technology Program (No. M201804). We would like to thank the TCGA research network for providing the data analyzed in this manuscript. The funding organizations had no roles in the study design, data collection, data analysis, interpretation and writing of the paper.

**Reference：**

1 Tong, Z. T. *et al.* EZH2 supports nasopharyngeal carcinoma cell aggressiveness by forming a co-repressor complex with HDAC1/HDAC2 and Snail to inhibit E-cadherin. *Oncogene* **31**, 583-594 (2012).

2 He, L. R. *et al.* High expression of EZH2 is associated with tumor aggressiveness and poor prognosis in patients with esophageal squamous cell carcinoma treated with definitive chemoradiotherapy. *International journal of cancer* **127**, 138-147 (2010).

3 Chen, J. *et al.* p53R2 as a novel prognostic biomarker in nasopharyngeal carcinoma. *BMC cancer* **17**, 846, doi:10.1186/s12885-017-3858-4 (2017).

4 Qian, C. N. *et al.* Preparing the "soil": the primary tumor induces vasculature reorganization in the sentinel lymph node before the arrival of metastatic cancer cells. *Cancer Res* **66**, 10365-10376, doi:10.1158/0008-5472.can-06-2977 (2006).

5 Zhu, W. *et al.* Overexpression of EIF5A2 promotes colorectal carcinoma cell aggressiveness by upregulating MTA1 through C-myc to induce epithelial-mesenchymaltransition. *Gut* **61**, 562-575 (2012).
